# Concerning a So-Called Dental College

**Published:** 1881-11

**Authors:** 


### ARTICLE VI.

### *Concerning a So-Called Dental College.*

(Report of Committee of the Wisconsin State Dental Association.)

"Your committee was entrusted one year ago to investigate the Wisconsin State Dental College," and after having procured considerable evidence, examined the articles of its corporation, and the laws of the State concerning its validity, find and herewith present legal opinion to confirm that in all the moral crookedness brought to light, that institution has not exceeded the limits prescribed by the Statutes. But that it has violated all precedents of professional integrity and uprightness by simply resolving itself for pure mercenary ends into a Diploma manufactory without any just regard for right or honor.

We herewith submit evidence, documents and legal opinion on this subject, and present the following preamble and resolutions, recommending their adoption, to wit:

*Whereas,* It has come to knowledge that certain men of doubtful professional standing have taken advantage of the Tax Law of the State of Wisconsin, Chapter 86, Revised Statutes of 1878, to prostitute to their private interests the privilege granted therein, and

*Whereas,* Ample proof in the shape of documents, circulars and signatures has been placed before us that an institution incorporated under the said law, calling itself the "Wisconsin Dental College," situated at Delavan, Walworth County, Wis., has been offering for sale, for money only, diplomas conferring the degree of D. D. S.,—Doctor of Dental Surgery—thereby cheapening and lowering the dignity and standing of the dental profession in the estimation of the public; aud endeavoring to place incompetent men before the public, on a par with men of known dental skill and experience; therefore

*Resolved*, That the Wisconsin State Dental Society pronounce such actions as unprofessional, dishonest, and so far as it succeeds, injurious to all concerned or affected by it, and therefore refuse to recognize or place any value upon all diplomas issued by said so called "Wisconsin Dental College."

*Resolved*, That the secretary be instructed to forward for publication a certified copy of this action of the Society."

This report was unanimously adopted, and a committee appointed to petition the Legislature to annul the charter of the so-called college at Delavan.

The Postmaster General has directed that no more circulars from the so-called Wisconsin Dental College be permitted to pass through the mails.

